# Spectrophotometric Online Detection of Drinking Water Disinfectant: A Machine Learning Approach

**DOI:** 10.3390/s20226671

**Published:** 2020-11-21

**Authors:** Sharif Hossain, Christopher W.K. Chow, Guna A. Hewa, David Cook, Martin Harris

**Affiliations:** 1 Scarce Resources and Circular Economy (ScaRCE), UniSA STEM, University of South Australia, Mawson Lakes, SA 5095, Australia; christopher.chow@unisa.edu.au (C.W.K.C.); guna.hewa@unisa.edu.au (G.A.H.); 2 Future Industries Institute, University of South Australia, Mawson Lakes, SA 5095, Australia; 3 Water Science Laboratory, South Australian Water Corporation, Adelaide, SA 5000, Australia; david.cook@sawater.com.au; 4 Operations & Water Quality, TRILITY, Adelaide, SA 5000, Australia; Martin.Harris@TRILITY.com.au

**Keywords:** chloramine, machine learning, online detection, spectral compensation, support vector regression, UV-Vis absorbance signatures

## Abstract

The spectra fingerprint of drinking water from a water treatment plant (WTP) is characterised by a number of light-absorbing substances, including organic, nitrate, disinfectant, and particle or turbidity. Detection of disinfectant (monochloramine) can be better achieved by separating its spectra from the combined spectra. In this paper, two major focuses are (i) the separation of monochloramine spectra from the combined spectra and (ii) assessment of the application of the machine learning algorithm in real-time detection of monochloramine. The support vector regression (SVR) model was developed using multi-wavelength ultraviolet-visible (UV-Vis) absorbance spectra and online amperometric monochloramine residual measurement data. The performance of the SVR model was evaluated by using four different kernel functions. Results show that (i) particles or turbidity in water have a significant effect on UV-Vis spectral measurement and improved modelling accuracy is achieved by using particle compensated spectra; (ii) modelling performance is further improved by compensating the spectra for natural organic matter (NOM) and nitrate (NO_3_) and (iii) the choice of kernel functions greatly affected the SVR performance, especially the radial basis function (RBF) appears to be the highest performing kernel function. The outcomes of this research suggest that disinfectant residual (monochloramine) can be measured in real time using the SVR algorithm with a precision level of ± 0.1 mg L^−1^.

## 1. Introduction

Conventional drinking water treatment processes consist of several stages to ensure treated water is safe for human consumption. In many countries, the final stage of treatment is the addition of a disinfectant to inactivate microorganisms in the water and to guard against recontamination and prevent the growth of biofilms [1]. Typically, chlorine and chloramines are the most widely used drinking water disinfectants [2,3]. In regional areas where disinfected water must travel to customers several hundred kilometres away, chloramines are ideal due to their greater stability compared with chlorine [1,2]. Chloramines have three different chemical forms: monochloramine (NH_2_Cl), dichloramine (NHCl_2_), and trichloramine (NCl_3_) [2,3,4]. Dichloramine and trichloramine have not been proven to be a suitable disinfectant because they are less stable than monochloramine and are reported to cause taste and odour issues [1,3]. Therefore, the term ‘chloramine disinfectant’ generally refers to monochloramine [2,3]. Continuous monitoring of monochloramine is required at desired points in a drinking water distribution system to ensure regulatory compliance [5].

Standard analytical methods of measuring the monochloramine residual concentration in aqueous solution include: (i) amperometric titration (standard method 4500-C1 D and ASTM method D 1253-86); (ii) DPD (N,N-diethyl-p-phenylenediamine) ferrous titrimetric (standard method 4500-C1 F); and (iii) DPD colorimetric method (standard method 4500-C1 G) [6]. Based on these methods, online analysers have been developed that can detect the monochloramine residual at the WTP and in the network. However, there are some drawbacks of these instruments. For example, the DPD colorimetric analyser requires chemical reagents to operate while the amperometric analyser needs frequent calibrations to ensure no drift of the zero calibration and regular replacement of the electrolyte for correct functioning [7]. Compared to this method, UV-Vis spectral detection provides flexibility, such as simple and reagent-free operation, rapid detection, and excellent repeatability [8]. UV-Vis spectral detection has been introduced in many areas, including wastewater, drinking water, river and sewer systems, disinfectant residual, and disinfection by-products (DBPs) [8,9,10,11,12,13,14].

Literature shows that chloramine species have strong light absorbance signatures in the ultraviolet wavelength range [15,16,17]. Spectral analysis by Gendel and Lahav [15] observed that monochloramine, dichloramine, and trichloramine have UV absorbance peaks at 243, 294, and 336 nm, respectively. Ferriol et al. [18] reported that monochloramine has an absorbance peak at a wavelength of 244 nm corresponding to a molar absorptivity of 458 mol^−1^ cm^−1^. On the other hand, Li and Blatchley [16] found that in aqueous medium, monochloramine has a maximum absorbance at the 245-nm wavelength with a molar absorptivity of 461 mol^−1^ cm^−1^. These studies confirmed that chloramine is sensitive to UV light and this criterion can be used to measure its concentration in a solution by applying the principle of the Beer–Lambert law [19].

Many studies indicated that machine learning has potential for the analysis of single or multi-wavelength spectral data [10,20,21,22,23]. For instance, using UV absorbance spectrometry in the 250–300-nm region, Kim et al. [24] used a multiple linear regression model to detect organic compounds in water. A partial least square (PLS) regression model was developed by Carré et al. [21] to establish a relationship between spectral data and total suspended solids (TSSs), turbidity, and chemical oxygen demand (COD) in reclaimed water. Wolf et al. [23] mapped the non-linear relationship between organic acid components and spectral data for online monitoring of anaerobic digestion processes in an industrial biogas plant. Some of the techniques they investigated for this mapping exercise included: (i) support vector machines (SVMs), (ii) linear discriminant analysis, (iii) generalised discriminant analysis (GerDA), (iv) random forest, and (v) neural networks. Similarly, using multi-wavelength absorbance spectrometry with a feed-forward neural network, Alves et al. [20] attempted to determine a river water quality index. In contrast, Chen et al. [22] assessed near-infrared (NIR) spectra using the least squares support vector machine (LSSVM) to develop a method for the quantitative determination of COD. Li and Hur [25] investigated the dynamics, fate, and distribution of dissolved organic matter (DOM) in various aquatic environments. They used Gaussian decomposition and correlation analysis to assess various spectral features, such as the differential and derivative spectra, spectral slopes, absorption ratios, absorption coefficient, etc. These are a few examples of machine learning applications in analysing and interpreting spectral data.

One major challenge in developing a spectrophotometry-based method for a specific water quality parameter is the interference caused by other light-absorbing substances. NOM and nitrate are major light-absorbing substances found in natural water bodies [19,26]. While during the water treatment process a considerable portion of NOM is removed, residual nitrate remains similar to the WTP inlet, affecting the measurement accuracy of the desired parameter. An appropriate spectral compensation may improve the measurement accuracy of that parameter. Many researchers have developed spectrophotometric methods to measure organic and nitrate concentrations [11,24,25,26]. Compared to UV light absorbance by organics and nitrate, monochloramine shows a relatively lower absorbance in the same wavelength range, so subtracting their absorbing contribution from the spectra will improve the measurement accuracy of monochloramine. Additionally, due to light scattering by suspended particles, turbidity in water causes a non-linear lifting of the spectrum, thereby reducing the measurement accuracy [27]. To minimise this effect, various particle compensation techniques, such as multiplicative scatter correction (MSC), theoretical model, and chemical and machine learning methods, have been developed [27,28,29]. In this study, a combination of particle, organic, and nitrate compensation was assessed.

The online spectrophotometric method of monochloramine detection is comparatively new, with little research completed in this area. Previous studies focused on applying the standard chemometric method using particle compensated spectra to relate spectral features with the monochloramine concentration. This study attempted to isolate the monochloramine spectra first by applying an additional spectral compensation for organic and nitrate. Hence, the objectives of the study were: (i) The development of spectral compensation to isolate the monochloramine spectra, and (ii) linking of the isolated spectra to amperometric monochloramine residual data using the machine learning algorithm.

**Figure 1:**
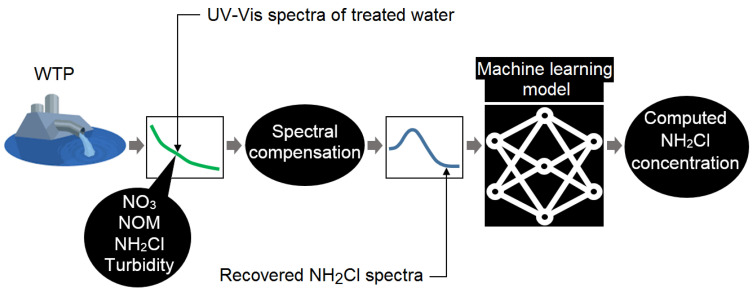
Schematic of the monochloramine detection method using UV-Vis spectra.

To date, to the authors’ best knowledge, no such method for online spectrophotometric measurement of monochloramine residual has been developed. This research showed that the regular field monitoring data of organic and nitrate levels could be an alternative to compensate the UV-Vis spectra for online detection of monochloramine. Improved modelling accuracy using such spectral compensation is the focus in this paper. The schematic of the proposed method is shown in Figure 1.

The remainder of this paper is organised as follows: Section 2 includes a description of the study area and relevant literature in this domain and methodology adopted in the research. Section 3 presents the results of the study. In Section 4, the machine learning modelling performances using different spectral compensation with different kernel functions are compared. Some limitations of the research are also discussed there and provide future lines of work. Section 5 concludes the paper.

## 2. Materials and Methods

### 2.1. Study Area

**Figure 2:**
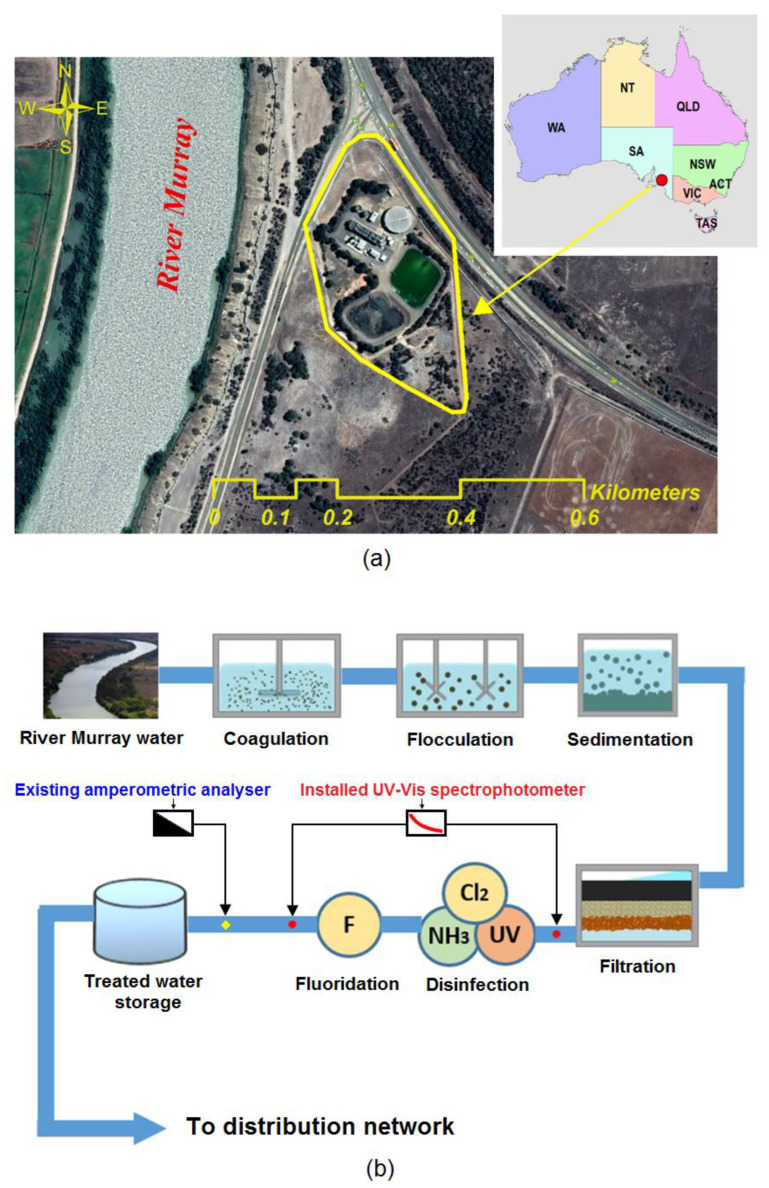
(**a**) Aerial view of Tailem Bend water treatment plant (WTP) and (**b**) Schematic of the water treatment process at Tailem Bend WTP and the installation location of the UV-Vis spectrophotometer (the spectrophotometer is fed water from two different sample points marked by the red dot in the figure).

The Tailem Bend drinking water distribution system is one of the major drinking water distribution systems operating in regional South Australia (Figure 2a). It is located at Tailem Bend township in South Australia, which is approximately 80 km southeast of Adelaide. The WTP collects water from River Murray and operates a conventional treatment process (coagulation → flocculation → sedimentation → filtration), with disinfection by UV irradiation and chloramination (Figure 2b). The treated water is then pumped into its distribution network, consisting of about a 143-km-long pipeline and several hundred kilometres of branch mains. Water quality at the WTP and multiple locations of the distribution network is monitored using various online-based devices.

The treated water has a varying level of turbidity, ranging between 0.04 and 0.12 nephelometric turbidity units (NTU) with a mean value of 0.08 NTU and standard deviation of 0.02 NTU. The upper and lower range of the monochloramine concentration during the study period was 5.5 and 3.0 mg L^‒1^ with a mean value of 4.3 mg L^‒1^ and standard deviation of 0.2 mg L^‒1^. Similarly, pH ranged between 7.8 and 9.3 with a mean value of 8.7 and dissolved organic carbon (DOC) ranged between 1.5 and 2.6 mg L^‒1^ with mean value of 2 mg L^‒1^. The standard deviations of the pH and DOC values were 0.3 and 0.3 mg L^‒1^, respectively.

At the WTP, an amperometric online chlorine analyser (Depolox 5, Wallace & Tiernan, Evoqua, Pittsburgh, PA, USA) is used to monitor the monochloramine residual, which is located after the disinfection and fluoride addition process (Figure 2b). The installed UV-Vis spectrophotometer and the online chlorine analyser are located close to each other to minimise the discrepancies in the hydraulic residence time (HRT) difference and the samples.

### 2.2. UV-Vis Spectrophotometric Device

The instrument used in this study was an online spectrophotometer probe from s::can Messtechnik GmbH, Austria that works on the principle of UV-Vis spectrometry. The more significant advantage of using spectrophotometric detection is that unlike many other online analysers, it does not require any chemical reagent to operate. The main component of the device consists of a stainless-steel body housing the UV-Vis spectrophotometer, which can be used either directly by placing it into the water sample or by attaching a sampling cell to the probe’s light path. Spectral data is obtained by a double beam of 256-pixel UV-Vis xenon light, which passes through the sample, with the absorbance value measured within wavelengths ranging from 200 to 750 nm with a 100-mm pathlength. In this study, the absorbance spectrum or fingerprint was measured every two minutes, with data stored in a computer connected to the probe.

At the WTP, the sampling cell of the spectrophotometer was fed from two different sample points: one was treated water prior to chloramination, which is termed as pre-chloraminated water; and the other was treated water after disinfection, which is termed as post-chloraminated water. Switching between the sources was controlled using an electronically controlled valve, and the duration of each source feeding to the sampling cell was set to 10 min.

### 2.3. Particle Interference on UV-Vis Spectrum and Compensation

Turbidity due to particles in water including silt, clay, organic and inorganic matter, and microscopic organisms may obstruct the transmittance of light, causing it to scatter, and thereby adding interference to the whole spectrum. The target compounds exist as dissolved species, so removal of particle absorbance is necessary to reduce interference. The standard procedure of measuring UV-Vis absorbance is to filter the sample through a 0.45-µm filter, so that filter retains the majority of these particles. Consequently, the corresponding spectrum is free from particle interference. For online spectrophotometric detection, physical filtering cannot be done easily as it is a slow process and cannot consistently deliver the required flow to the device. Therefore, the unfiltered spectrum obtained results in light scattering that need to be corrected to get the absorbance by dissolved compounds in the water matrix. This process is known as turbidity or particle compensation. Several particle compensation techniques exist in the literature [27,28,29,30]. The software equipped with the spectrophotometer has a built-in function to do this operation for different water types (i.e., drinking water, wastewater, etc.).

### 2.4. Support Vector Regression

**Figure 3:**
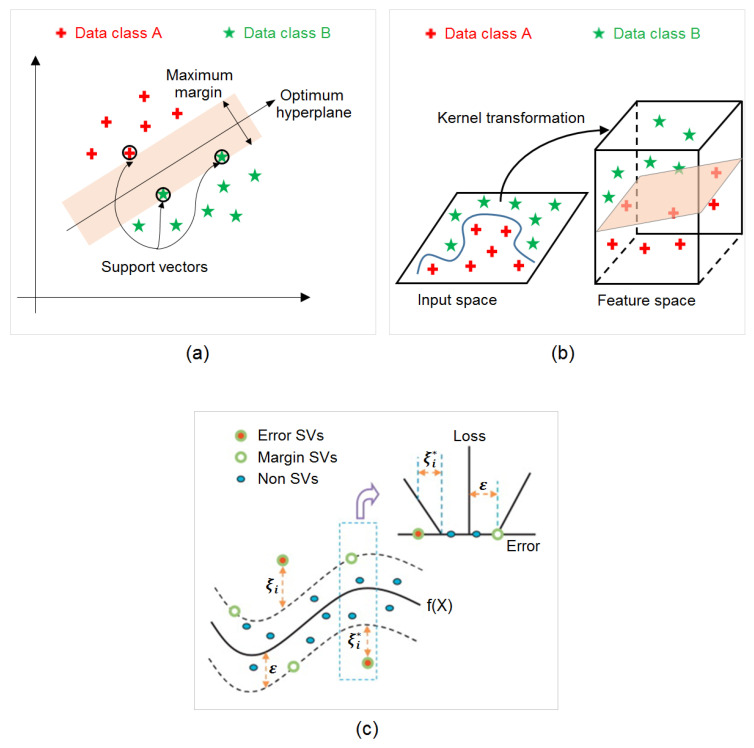
Concepts of the support vector classification (SVC) algorithm for (**a**) linear separable cases, (**b**) non-linear separable cases with kernel transformation, and (**c**) non-linear SVR (adapted from Raghavendra and Deka [36]).

Support vector machines (SVMs) are a popular machine learning algorithm introduced by Vapnik and other researchers [31,32,33]. The concept originated from statistical learning theory for solving a constrained quadratic problem where the convex objective function for optimisation is represented by a combination of loss function and a regularisation term [34]. The two most common applications of SVMs are support vector classification (SVC) and support vector regression (SVR). For the classification problems, the objective is to find the optimal separating hyperplane that maximises the margin of the training data. A hyperplane can be defined as a boundary that separates various data classes. In an n- dimensional Euclidean space, the hyperplane is a subset of that space with dimension n-1 that divides the space into two disconnected parts. Data points that are closer to the hyperplane are called support vectors (SVs). Figure 3a shows an optimal hyperplane with a maximising margin between two classes of data. Although the concept of the SVC algorithm was originally based on binary classification, it can be extended to multi-class classification problems by combining a series of binary classifiers [35].

However, in many cases, data may not be linearly separable. Under such a condition, SVC uses a kernel trick (with the details discussed afterwards) to map the data in a high-dimensional space where linear separation is possible [34,36,37,38]. There are theorems that guarantee the existence of such kernel functions under certain conditions [32,33,39]. This is shown in Figure 3b, where two classes of data, A and B, are linearly inseparable in the two-dimensional input space, but after transforming to three-dimensional feature space, the separation becomes possible.

In SVM for a regression problem, the objective is to fit a model to predict a quantity for the future. Thus, the data points are expected to be distributed closely around the regression line except that an epsilon (*ε*) range is defined from both sides of the hyperplane where the regression function is considered to be insensitive (Figure 3c). Errors smaller than *ε* do not matter while if they are greater than *ε*, they are of concern. The theory behind the SVR algorithm can be found in many studies [31,32,33,34,36,38,39]. Based on this literature, a description is provided below.

Consider a dataset $\left\{\left(x_{1},y_{1}\right),\left(x_{2},y_{2}\right),\ldots \ldots ,\left(x_{l},y_{l}\right)\right\}\subset X\times R$, SVR tries to fit a function f(x) for all the training data that has at most *ε* deviation from the actually obtained targets $y_{i}$ and at the same time keeping it as flat as possible (Figure 3c). In the case of linear functions, the equation can be written as:

(1) f(x)=〈ω,x〉+b  with ω∈X,   b∈R,

where the symbol 〈.,.〉 in Equation (1) represents the dot product in X and the parameter ω represents a dimensional weight vector that controls the flatness of the function. The smaller the value of ω, the flatter the function. This can be achieved by minimising the Euclidean norm ${\left\|\omega \right\|}^{2}$. Therefore, it can be considered as a convex optimisation problem by requiring:

(2) $$[\begin{array}{l} \mathrm{minimise}\;\frac{1}{2}{\left\|\omega \right\|}^{2}\\ \mathrm{subject}\;\mathrm{to}\;\{\begin{array}{c} y_{i}-\langle \omega ,x_{i}\rangle -b\leq \varepsilon \\ \langle \omega ,x_{i}\rangle +b-y_{i}\leq \varepsilon \end{array} \end{array}.$$

The solution of Equation (2) is feasible in cases where the function (f) actually exists and approximates all pairs $\left(x_{i},y_{i}\right)$ with *ε* precision. Otherwise, slack variables $\xi_{i},\xi_{i}^{*}$ are introduced to allow some error to cope with infeasible constraints of the optimisation problem. Thus, the optimisation equation can be rewritten as:

(3) $$[\begin{array}{l} \mathrm{minimise}\;\frac{1}{2}{\left\|\omega \right\|}^{2}+C\overset{l}{\underset{i=1}{\sum }}\left(\xi_{i}+\xi_{i}^{*}\right)\\ \mathrm{subject}\;\mathrm{to}\;\{\begin{array}{c} y_{i}-\langle \omega ,x_{i}\rangle -b\leq \varepsilon +\xi_{i}\\ \langle \omega ,x_{i}\rangle +b-y_{i}\leq \varepsilon +\xi_{i}^{*} \end{array} \end{array}.$$

The constant C > 0 indicates the trade-off between the flatness of the function (f) and the amount of maximum deviations permitted over *ε.* The optimisation problem in Equation (3) can be solved by constructing a dual problem, where the aim is to maximise the objective function in terms of the dual variables under the derived constraints on the dual variables. The first step is to construct a Lagrange function by adding the constraints to the objective function:

(4) $$[\begin{array}{l} L=\frac{1}{2}{\left\|\omega \right\|}^{2}+C\overset{l}{\underset{i=1}{\sum }}\left(\xi_{i}+\xi_{i}^{*}\right)-\overset{l}{\underset{i=1}{\sum }}\alpha_{i}\left(\varepsilon +\xi_{i}-y_{i}+\langle \omega ,x_{i}\rangle +b\right)\;\\ \;\;\;\;\;\;\;\;\;\;\;\;-\overset{l}{\underset{i=1}{\sum }}\alpha_{i}^{*}\left(\varepsilon +\xi_{i}^{*}+y_{i}-\langle \omega ,x_{i}\rangle -b\right)-\overset{l}{\underset{i=1}{\sum }}\eta_{i}\xi_{i}+\eta_{i}^{*}\xi_{i}^{*} \end{array}.$$

The dual variables in Equation (4) need to fulfil the conditions $\alpha_{i},\;\alpha_{i}^{*},\eta_{i},\;\eta_{i}^{*}\geq 0$. It follows from the saddle point definition that the partial derivatives of *L* in terms of the primal variables $\left(\omega ,b,\xi_{i},\xi_{i}^{*}\right)$ have to vanish to reach the optimal condition:

(5) $$\frac{\partial L}{\partial b}=\overset{l}{\underset{i=1}{\sum }}\left(\alpha_{i}^{*}-\alpha_{i}\right)=0,$$

(6) $$\frac{\partial L}{\partial \omega }=\omega -\overset{l}{\underset{i=1}{\sum }}\left(\alpha_{i}-\alpha_{i}^{*}\right)x_{i}=0,$$

(7) $$\frac{\partial L}{\partial \xi_{i}^{*}}=C-\alpha_{i}^{*}-\eta_{i}^{*}=0.$$

Substituting Equations (5) to (7) into Equation (4) yields the following dual optimisation problem:

(8) $$[\begin{array}{l} \mathrm{maximise}\left\{-\frac{1}{2}\overset{l}{\underset{i,j=1}{\sum }}\left(\alpha_{i}-\alpha_{i}^{*}\right)\left(\alpha_{j}-\alpha_{j}^{*}\right)\langle x_{i},x_{j}\rangle -\varepsilon \overset{l}{\underset{i=1}{\sum }}\left(\alpha_{i}+\alpha_{i}^{*}\right)+\overset{l}{\underset{i=1}{\sum }}y_{i}\left(\alpha_{i}-\alpha_{i}^{*}\right)\right\}\\ \mathrm{subject}\;\mathrm{to}\overset{l}{\underset{i=1}{\sum }}\left(\alpha_{i}-\alpha_{i}^{*}\right)=0\;\mathrm{and}\;\alpha_{i},\alpha_{i}^{*}∊\left[0,C\right] \end{array}.$$

The dual variables $\eta_{i}\eta_{i}^{*}$ do not appear in Equation (8) because through Equation (7) they have been eliminated. Equation (6) can be rewritten as:

(9) $$\omega =\overset{l}{\underset{i=1}{\sum }}\left(\alpha_{i}-\alpha_{i}^{*}\right)x_{i}.$$

Therefore:

(10) $$f\left(x\right)=\overset{l}{\underset{i=1}{\sum }}\left(\alpha_{i}-\alpha_{i}^{*}\right)\langle x_{i},x\rangle \;+b.$$

This is the support vector (SV) expansion for the function (f). Equation (10) indicates that the term ω in Equation (1) can be represented by a linear combination of the training patterns $x_{i}$, while b can be computed by applying the Karush–Kuhn–Tucker conditions [40], which states that the product between dual variables and constraints has to vanish at the optimal solution. This means:

(11) $$[\begin{array}{l} \alpha_{i}\left(\varepsilon +\xi_{i}-y_{i}+\langle \omega ,x_{i}\rangle +b\right)=0\\ \alpha_{i}^{*}\left(\varepsilon +\xi_{i}^{*}+y_{i}-\langle \omega ,x_{i}\rangle -b\right)=0 \end{array},$$

and:

(12) $$[\begin{array}{l} \left(C-\alpha_{i}\right)\xi_{i}=0\\ \left(C-\alpha_{i}^{*}\right)\xi_{i}^{*}=0 \end{array}.$$

Based on these conditions, some conclusions can be made. Firstly, the ε-insensitive tube around the function (f) does not include samples $\left(x_{i},\;y_{i}\right)$ with corresponding $\alpha_{i}^{*}=C$. Secondly, a set of dual variables $\alpha_{i},\;\alpha_{i}^{*}$, both simultaneously nonzero, does not exist as it requires non-zero slacks in both directions; therefore, $\alpha_{i}\alpha_{i}^{*}=0$. Finally, for $\alpha_{i}^{*}\in \left(0,C\right)$ results in $\xi_{i}^{*}=0$, and the second factor in Equation (11) has to vanish. Thus, b can be computed as:

(13) $$[\begin{array}{l} b=y_{i}-\langle \omega ,x_{i}\rangle -\varepsilon \;\;\;\;\;\mathrm{for}\;\;\alpha_{i}\in \left(0,C\right)\\ b=y_{i}-\langle \omega ,x_{i}\rangle +\varepsilon \;\;\;\;\;\mathrm{for}\;\alpha_{i}^{*}\in \left(0,C\right) \end{array}.$$

The SVR algorithm can be extended to non-linear functions through mapping of the data (*X*) to another space, called feature space (*F*), by applying a transformation function ϕ:X→F and then using the standard SVR algorithm [34,39]. Thus, for a non-linear case, the optimisation problem becomes about finding the flattest function in the feature space instead of the input space.

The kernel function can be defined as a linear dot product in the feature space. It can be shown that for certain mappings ϕ, kernel functions $k\left(x_{i},\;x_{j}\right)=\langle \phi \left(x_{i}\right),\;\phi \left(x_{j}\right)\rangle$ exist [32,33,39]. The functions $k\left(x_{i},\;x_{j}\right)$ have to satisfy Mercer’s condition [34,39]. Since solving the dual problem in the SV algorithm depends on the values of the dot product, a kernel function can be used instead of ϕ. Therefore, the algorithm can be rewritten as:

(14) $$[\begin{array}{l} \mathrm{maximise}\left\{-\frac{1}{2}\overset{l}{\underset{i,j=1}{\sum }}\left(\alpha_{i}-\alpha_{i}^{*}\right)\left(\alpha_{j}-\alpha_{j}^{*}\right)k(x_{i},x_{j})-\varepsilon \overset{l}{\underset{i=1}{\sum }}\left(\alpha_{i}+\alpha_{i}^{*}\right)+\overset{l}{\underset{i=1}{\sum }}y_{i}\left(\alpha_{i}-\alpha_{i}^{*}\right)\right\}\\ \mathrm{subject}\;\mathrm{to}\overset{l}{\underset{i=1}{\sum }}\left(\alpha_{i}-\alpha_{i}^{*}\right)=0\;\mathrm{and}\;\alpha_{i},\alpha_{i}^{*}∊\left[0,C\right] \end{array}.$$

The function (f) can now be expressed as:

(15) $$f\left(x\right)=\overset{l}{\underset{i=1}{\sum }}\left(\alpha_{i}-\alpha_{i}^{*}\right)k\left(x_{i},x\right)+b.$$

The SVR modelling accuracy can be improved with the right choice of kernel function as different kernel functions have different mapping capabilities. The four kernel functions given in Equations (16) to (19) are most commonly used in the SVR algorithm [22,35,36,37,38,39,41]:

(16) $$(i)\;\mathrm{Linear}:\;k\left(x_{i},x_{j}\right)=\left(x_{i}^{T}x_{j}\right),$$

(17) $$(\mathrm{ii})\;\mathrm{Polynomial}:\;k\left(x_{i},x_{j}\right)={\left(\Upsilon x_{i}^{T}x_{j}+r\right)}^{d},$$

(18) $$(\mathrm{iii})\;\mathrm{RBF}:\;k\left(x_{i},x_{j}\right)=exp\left(-\Upsilon \|x-{y\|}^{2}\right),\;\;\;\;\Upsilon >0,$$

(19) $$(\mathrm{iv})\;\mathrm{Sigmoid}:\;k\left(x_{i},x_{j}\right)=tanh\left(\Upsilon x_{i}^{T}x_{j}+r\right),$$

where $x_{i}^{T}$ is the transpose of $x_{i}$, *r* is a constant term, d is the polynomial order, and Υ is a RBF kernel parameter that controls the spread of the data while transforming to higher dimensions.

### 2.5. Methodology

**Figure 4:**
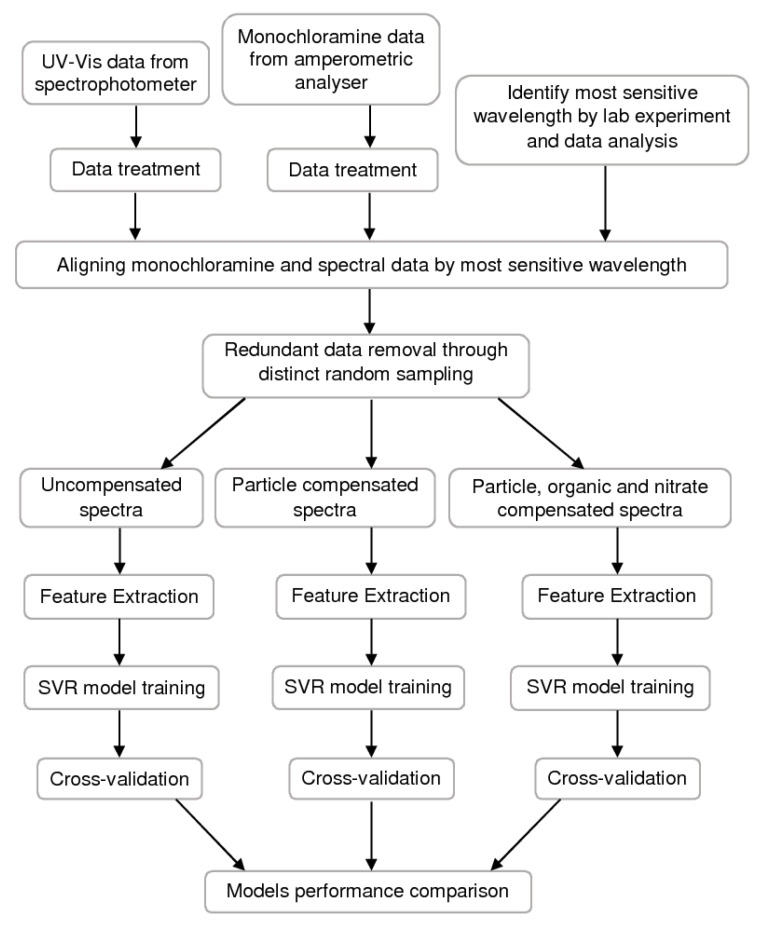
Work methodology.

The systematic procedure adopted in this research is presented in Figure 4.

**Figure A1:**
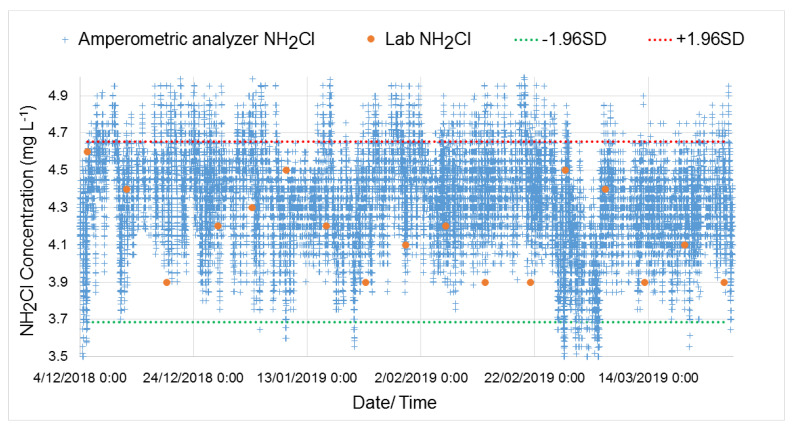
Comparison of online monochloramine analyser data with lab data (green and red dotted line indicate 95% confidence interval for lower and upper limit).

Light absorbance data from a spectrophotometer and monochloramine concentration data from an amperometric analyser were collected during the period from December 2018 to March 2019 and processed for further analysis. The validation of the amperometric analyser data is given in Appendix A, Figure A1. Missing values in the data were estimated by linear interpolation. Outliers were checked using the modified z-score method [42,43,44] and removed from the data. This method is expressed by the following equation:

(20) $$M_{i}=\frac{0.6745\left(x_{i}-\tilde{x}\right)}{MAD},$$

where $M_{i}$ is the modified z-score, and $x_{i}$ and $\tilde{x}$ are the *i*th ordinate and median of a feature vector, respectively. The median absolute deviation (MAD) is given by:

(21) $$MAD=median\left|x_{i}-\tilde{x}\right|.$$

The modified *z*-score method is more robust than the standard *z*-score method. This is because while calculating the standard *z*-score, the arithmetic mean and standard deviations are used. Therefore, the computed *z*-score can be significantly affected by a few extreme values or by even a single extreme value. This does not happen in the case of the modified *z*-score as it uses the median value instead of the mean. According to many researchers, including Iglewicz and Hoaglin [44], a modified *z*-score greater than 3.5 can be considered an outlier.

To properly align a spectral signal with monochloramine data, the MATLAB (The MathWorks Inc., Natick, MA, USA) interactive file brushing tool was used. Firstly, the correlation of the absorbance at various wavelengths to the monochloramine concentration was assessed. The Pearson correlation was calculated using the following formula:

(22) $$\mathrm{Pearson}’s\;r=\frac{n\sum_{i=1}^{n}(R_{i}\cdot P_{i})-(\sum_{i=1}^{n}R_{i})\cdot (\sum_{i=1}^{n}P_{i})}{\sqrt{\left(n\sum_{i=1}^{n}R_{i}{}^{2}-{\left(\sum_{i=1}^{n}R_{i}\right)}^{2}\right).\left(n\sum_{i=1}^{n}P_{i}{}^{2}-{\left(\sum_{i=1}^{n}P_{i}\right)}^{2}\right)}},$$

where $R_{i},\;P_{i}$ are the *i*th data points from the spectra and monochloramine concentration, respectively, and n is the total number of data points.

**Figure 5:**
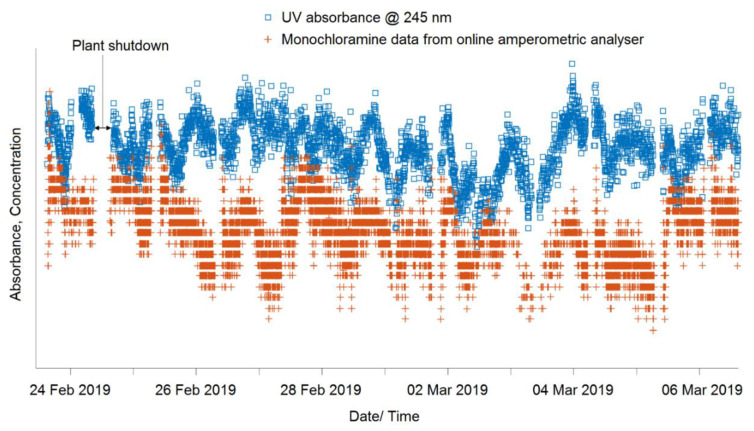
Alignment of monochloramine data with UV-Vis spectra.

The wavelength corresponding to the maximum correlation was considered as the representative wavelength, which was at 245 nm. Spectral analysis of the monochloramine solution at different levels using a benchtop laboratory spectrophotometer also indicated a peak at 245 nm. This wavelength and monochloramine concentration data were plotted in a single graph in MATLAB using appropriate scale settings. A portion of the whole time series is shown in Figure 5, where some gaps were identified due to plant shutdown. For each segment, cross-correlation was considered to determine the alignment appropriateness. These methods of alignment served two purposes: (i) identifying if there is any hydraulic residence time (HRT) between the data sources, and (ii) if there is any clock time difference between them. The alignment corresponding to the maximum cross-correlation was considered as the appropriate alignment. The whole time series was aligned segment by segment.

It was found that the historic monochloramine data from the amperometric analyser contained a considerable number of numerical values that were repeated several times in the data. This is due to the different protocol settings of the data historian software. To overcome this issue, a random sampling from monochloramine data was done in such way that each numerical value could not appear more than one time. This ensured a unique model training while the employing machine learning algorithm. *R* codes were utilised to perform distinct random sampling several million times. For each random sampling, the goodness-of-fit between the monochloramine data and spectral time-series was assessed and the numerical seed that provided the maximum match was considered as the appropriate seed in random sampling. The resulting data were used in the model.

Particle compensation is a vital component to be considered while analysing any light-absorbing spectral data. In this study, particle compensation was completed by using the offline spectral data processing tool that accompanies the spectrophotometer. It offers particle compensation for various water types, such as drinking water, wastewater, river water, etc., where the drinking water category was selected for the compensation.

**Figure 6:**
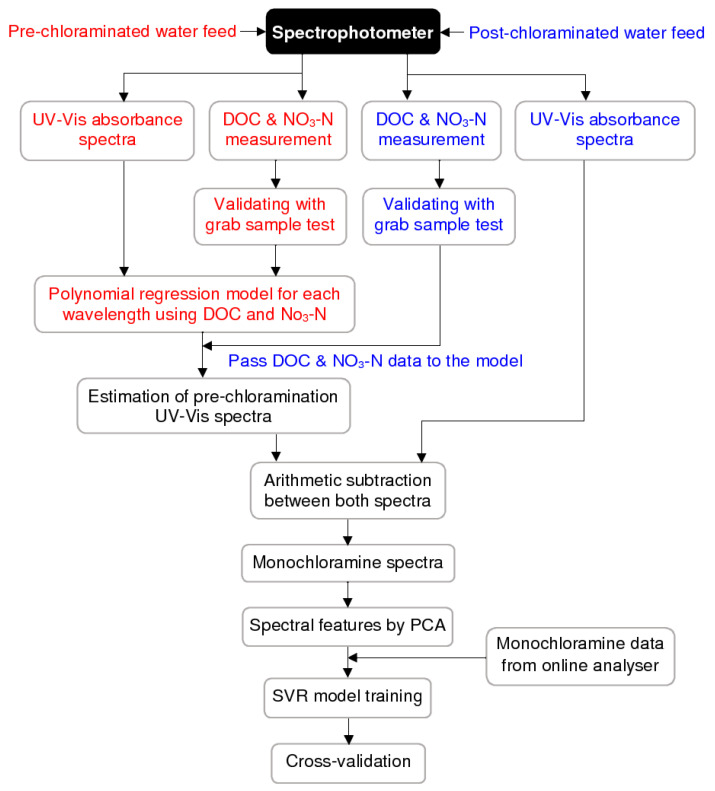
Procedure for separating monochloramine spectra (red colour refers to the process related to pre-chloraminated water and blue colour for post-chloraminated water).

NOM is the dominant light-absorbing component in water and can interfere with monochloramine spectra [19,26]. Additionally, the presence of nitrate (expressed as NO_3_-N) may absorb UV light [26]. A compensation for organic and nitrate was applied here to separate monochloramine spectra from the recorded post-chloramination spectra. The objective was to determine whether separating the monochloramine spectra and training the SVR model using them improved the accuracy. The detailed procedure of separating the monochloramine spectra is shown in Figure 6.

**Figure 7:**
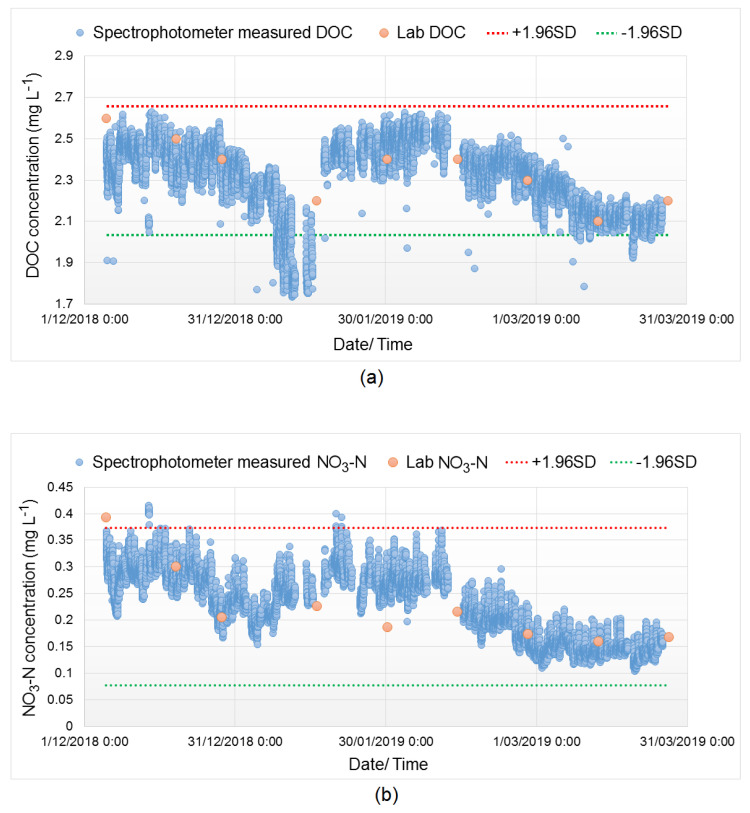
Calibration of the spectrophotometer using lab data for (**a**) DOC measurement and (**b**) NO_3_-N measurement (red and green dotted line indicate the 95% confidence interval for the upper and lower limit).

As shown in Figure 6, the spectra fingerprints of both pre-chloraminated and post-chloraminated water at the WTP were obtained by a single spectrophotometer probe that gives measurement of a range of water quality parameters. The spectrophotometric module was calibrated to match the DOC and NO_3_-N measurements with lab-measured values. Figure 7 shows the calibration of DOC and NO_3_-N parameters, where trends indicate a good level of agreement between these data.

It was assumed that spectral configuration of pre-chloraminated water was mainly governed by DOC and NO_3_-N. Therefore, using these parameters, a polynomial regression model was developed for each absorbing wavelength by utilising *R* programming codes. A fourth-order polynomial function was used to model the spectra. During the chloramination process at the WTP, oxidation reactions may occur while mixing ammonia and chlorine to water, potentially causing DOC and NO_3_-N concentrations to change. These were assumed to have a minor effect because the source water location to the spectrophotometer was immediately after chloramination. Therefore, it was assumed that the DOC and NO_3_-N concentrations in the post-chloraminated water created similar spectra as pre-chloraminated water spectra while passing through the regression model. Direct subtraction was not considered to be accurate because both pre-chloraminated and post-chloraminated water was monitored using a single spectrophotometer, so they had different timestamps while spectral measurements were taken.

Machine learning modelling accuracy can be impacted by multi-collinearity problems if a high correlation exists between feature variables [45]. It has been found that light absorbance for a specific wavelength is highly correlated to the neighbouring wavelengths, and correlation gradually decreases to far wavelengths. Therefore, to avoid redundancy in the model training, principal components were extracted and used in modelling rather than using absorbance values directly. Moreover, the use of principle components can ensure maximum performance of the machine learning algorithm as the data size is significantly reduced by principle component analysis (PCA). It has been found that factors producing eigenvalues greater than 0.01 can explain 99.9% of the variance of the data, and therefore these factors were considered as feature variables to build up the model. 

The SVR method in Unscrumbler X (CAMO software, Oslo, Norway) was used to build up the model and its performance was evaluated under four different kernel functions: (i) linear, (ii) polynomial, (iii) RBF, and (iv) sigmoid. Among the SVR parameters, ε controls the width of the hyperplane. A comparatively larger value of ε indicates fewer support vectors are selected in the modelling, resulting in more flat estimates by the model. According to Mattera and Haykin [46], an ε value that causes the number of support vectors to be approximately 50% of the data length can be considered a good choice. In this study, ε was selected as 0.01, causing approximately 50% of the support vectors of the data.

*C* is an SVC learner parameter and it represents the penalty of misclassifying a data point. Comparatively smaller *C* values indicate some misclassification of data will be encountered by the classifier. In contrast, a more substantial value of *C* represents the classifier will be heavily penalised for misclassified data points. Apart from *C*, the parameter Υ also need to be optimised. A low value of Υ indicates a very broad decision region whereas a high value creates islands of decision boundaries around data points. The value of Υ can be estimated as $\Upsilon =\frac{1}{2\sigma^{2}}$, where σ represents the Gaussian noise level of the standard deviation [34]. Both C and Υ values were obtained by using the built-in grid search method in Unscrumbler X while a third-degree polynomial function was used in modelling with the polynomial kernel.

Among the various methods used in model validation in machine learning, hold-out validation and k-fold cross-validation are widely used. In the first case, data is required to split into a training set and a testing set. However, dividing the original data can cause information loss, thereby increasing the error induced by bias. Therefore, to minimise the error, a 10-fold cross-validation procedure was adopted. As a general rule, a 5-fold or 10-fold cross-validation has been empirically shown to ensure that the error estimate suffers neither high bias nor high variance. Goodness-of-fit between the reference and model predicted was evaluated by the coefficient of determination (R-square), and root mean square error (RMSE) [36,38,47] as given by the following formula:

(23) $$\mathrm{Coefficient}\;\mathrm{of}\;\mathrm{determination}\;\left(R-\mathrm{square}\right)\;=\;\left(1-\frac{\sum_{i=1}^{n}{\left(R_{i}-P_{i}\right)}^{2}}{\sum_{i=1}^{n}{\left(R_{i}-\bar{R}\right)}^{2}}\right),$$

(24) $$\mathrm{Root}\;\mathrm{mean}\;\mathrm{square}\;\mathrm{error}\;\left(\mathrm{RMSE}\right)\;=\;\sqrt{\frac{\sum_{i=1}^{n}{\left(P_{i}-R_{i}\right)}^{2}}{n}},$$

where $R_{i}$ and $P_{i}$ are the reference and predicted values of the monochloramine concentration, respectively, n is the total number of data points, and $\bar{R}$ is the mean of the reference values. The value of R-square ranges from 0 to 1, where 1 means perfect fit and 0 means no fit at all. RMSE has no scale, but a best-fitted model will encounter a low RMSE value.

Data normalisation is an integral part of machine learning. To minimise bias, all feature variables in the data were normalised before SVR analysis. The purpose is to bring their values to a common scale so that the model training becomes less sensitive to the scale of features as regularization behaves differently for different scaling. Properly scaled feature variables can ensure convergence of the SVR algorithm. In this study, data were scaled to −1 to +1 by using the following formula:

(25) $$x^{\prime }=\;\frac{x_{i}-µ}{\max\left|x_{i}-µ\right|},$$

where $x^{\prime }$ is the normalised data, $x_{i}$ is the *i*th ordinate of a feature vector, µ is the mean value, and “max” represents the maximum value.

## 3. Results

### 3.1. Monochloramine Peak Absorbance Wavelength Detection and Particle Compensation

**Figure 8:**
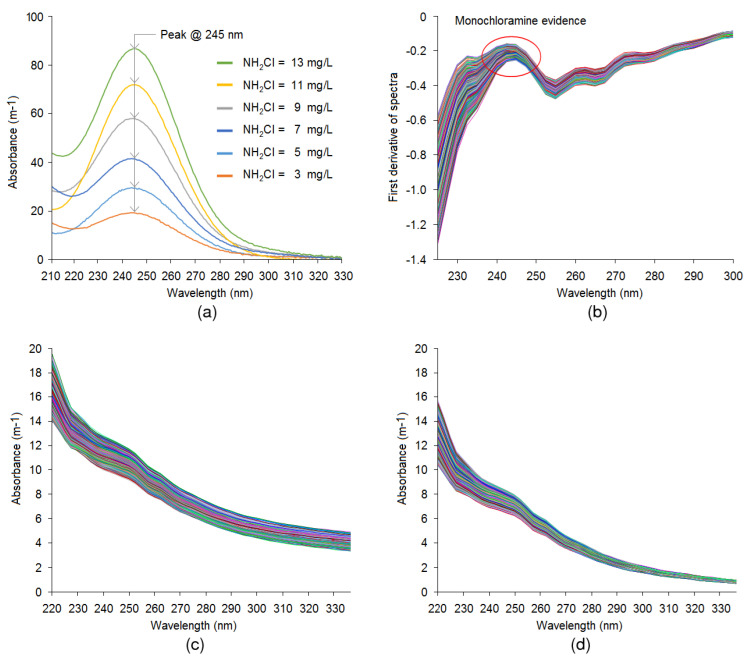
(**a**) Monochloramine spectra in Milli-Q water with different levels of concentration. (**b**) First derivative of spectra. (**c**) Uncompensated spectra, and (**d**) particle compensated spectra.

The spectra fingerprint of monochloramine, hence the peak absorbance wavelength, was determined in ultrapure water from a Milli-Q water purification system (Millipore, Molsheim, France) using a benchtop laboratory spectrophotometer. The resulting spectra between 210 and 330 nm corresponding to various monochloramine levels are presented in Figure 8a, indicating that absorbance increases as concentration increases and peak absorbance appears at about 245 nm for all concentrations. The remaining portion of the spectra is comparatively flat. For all monochloramine solutions, pH was kept constant, at approximately 8.5, to avoid spectral shifting and to match with the operational pH practised at the Tailem Bend WTP. In Figure 8a, it is seen that the starting absorbance of some spectra with a low concentration of monochloramine is comparatively higher than the spectra with a high concentration of monochloramine. This is due to a relatively high amount of dichloramine formation during the preparation of the monochloramine solution.

Unlike Milli-Q water, treated water at the WTP contains several light-absorbing substances with peaks at different wavelengths. Therefore, derivative spectra were derived from online data from a spectrophotometer to identify the location where the major peak appears. Figure 8b shows the first derivative of spectra within the 225–300 nm region, which indicates a sudden slope change marked by the red circle in the figure. This is caused by monochloramine spectra with a peak at about 245 nm. It can be seen in Figure 8b, a minor peak in the derivative spectra appears between 260 and 280 nm. According to Roccaro et al. [48], the derivative spectra at 272 nm may relate to chlorinated disinfection by-products and precursors. The remaining region of the derivative spectra is comparatively flat.

Furthermore, a Pearson’s correlation analysis was performed between the spectral absorbance at various wavelengths and monochloramine data from the amperometric analyser. The results indicated significant correlation between the two data sets at the 0.01 level and maximum correlation occurred at about 245 nm with a correlation coefficient value of 0.54. Therefore, this wavelength was used to align both data sets.

**Figure A2:**
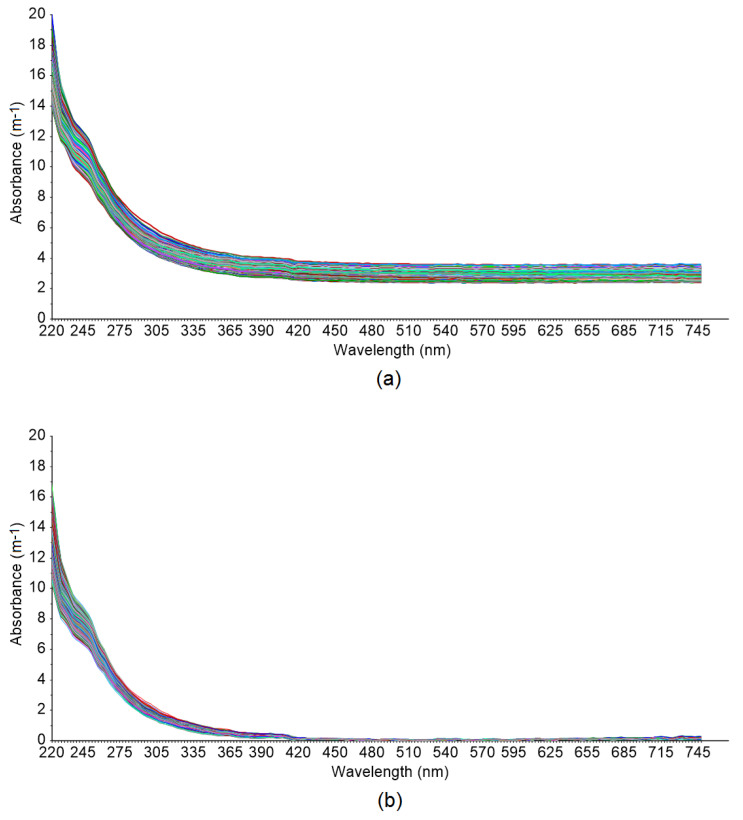
Post chloramination spectra at the WTP: (**a**) Uncompensated spectra and (**b**) particle compensated spectra (absorbance is relatively high in uncompensated spectra due to particle interference and after particle compensation absorbance is significantly reduced).

Figure 8c shows the uncompensated spectra obtained from the UV-Vis spectrophotometer while Figure 8d shows the corresponding particle compensated spectra obtained by processing uncompensated spectra using the spectrophotometer’s built-in particle compensation tool. For better viewing, wavelengths between 220 and 330 nm are displayed in the figure while the full wavelength spectra are provided in Appendix A, Figure A2. It is evident from these figures that uncompensated spectra show a relatively higher absorbance than particle compensated spectra as the light absorbance by the particle is removed through particle compensation. The difference between the two is the compensation due to the light-scattering effect. The accuracy of particle compensated spectra was verified by accessing the correlation of the absorbance at various wavelengths to monochloramine data from the amperometric analyser. The analysis indicated that after correcting the spectra through particle compensation, the correlation coefficient improved from 0.54 to 0.62.

### 3.2. Spectral Compensation for Organic and Nitrate

**Figure 9:**
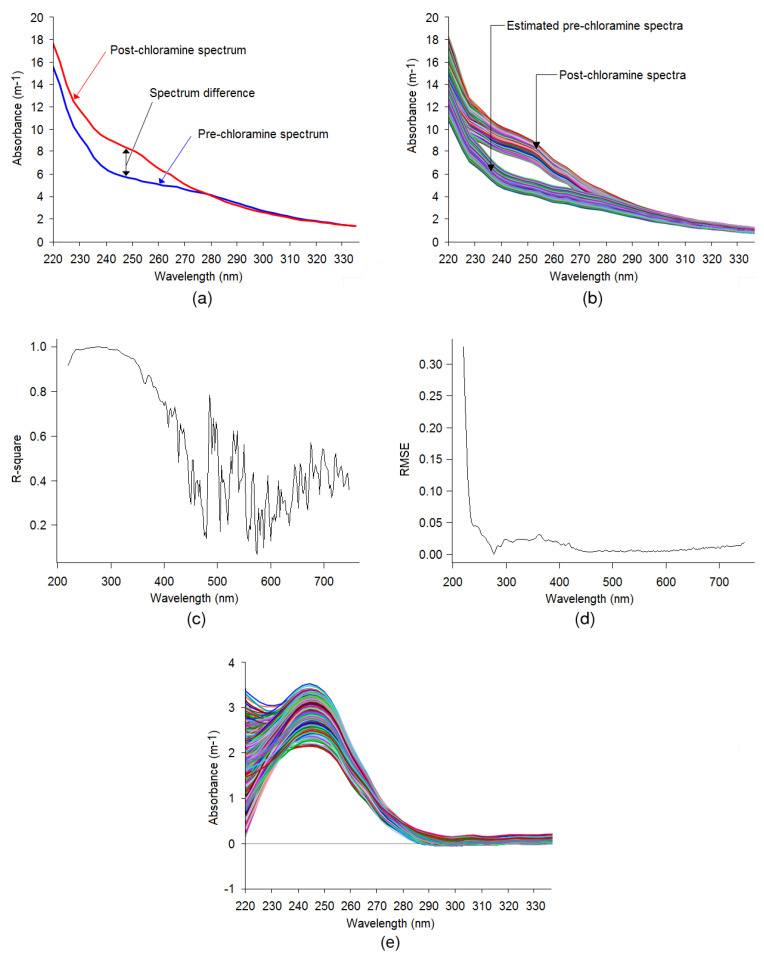
(**a**) Typical pre and post chloraminated spectra recorded at the WTP. (**b**) Comparison of estimated pre-chloraminated spectra and original post-chloramination spectra. (**c**) Coefficient of determination values in polynomial model fitting for various wavelengths. (**d**) Root mean square error (RMSE) values for polynomial fit. (**e**) Estimated DOC and NO_3_-N compensated (NH_2_Cl) spectra.

**Figure A3:**
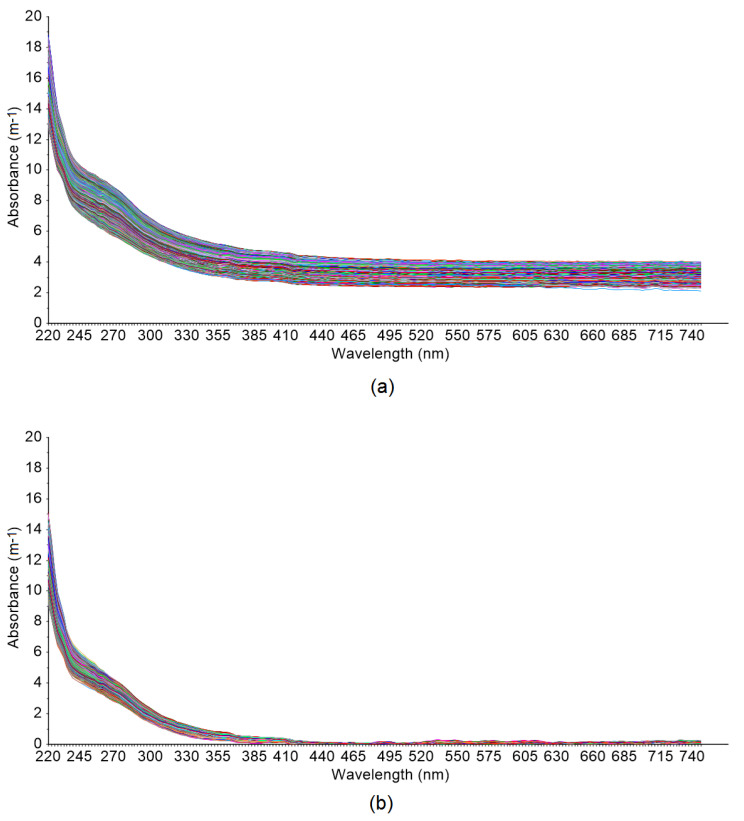
Pre chloramination spectra at the WTP: (**a**) Uncompensated spectra and (**b**) particle compensated spectra (Figure A3b was considered to develop polynomial regression model).

A comparison of the typical pre-chloraminated and post-chloraminated water spectrum recorded at the WTP with particle compensation within the 220–330 nm range is given in Figure 9a while the full UV-Vis range of wavelengths is available in Appendix A, Figure A2 and Figure A3. In the figure, it is evident that after adding monochloramine, the light absorbance by water is increased in between wavelengths of 220 and 280 nm. The remaining region of the spectra is overlaid. Figure 9b shows the post-chloramination spectra and estimated pre-chloramination spectra in the same plot, which clearly indicates the absorbance by the estimated spectra is comparatively lower within wavelengths from 220 to 280 nm. Moreover, the remaining regions of the two spectra were overlaid, closely resembling Figure 9a.

Figure 9c shows the accuracy of the polynomial model for each spectral wavelength measured in terms of the coefficient of determination. The R-square values indicate that the DOC and NO_3_-N correlations to spectral wavelengths in between 220 and 400 nm are maximised with low variability, while for the rest of the wavelengths, the correlation is irregular. Therefore, the wavelengths within 220 to 400 nm mainly contribute to estimating the spectral configuration of pre-chloraminated water. The RMSE values in Figure 9d indicate that the starting RMSE is comparatively higher and gradually decreases to far wavelengths. This is due to the relatively high absorbance value at the starting wavelength as the molar absorptivity increases with a decreasing wavelength, thereby encountering a comparatively high residual error in the model fitting. From 400 nm and greater wavelengths, the RMSE values are close to zero because the absorbance in this region is very low. Hence, the residuals are very low in the model fitting as compared to spectra in the 220–400 nm region. The numeric data for Figure 9c,d are available in Appendix B, Table A1. As can be seen in Figure 9a, after the addition of monochloramine disinfectant, spectral changes occurred between wavelengths of 220 and 280 nm. The polynomial regression model performance in terms of R-square in that region using DOC and NO_3_-N data varies from 0.92 to 0.99 (Table A1, Appendix B). The R-square value close to 1 indicates that organic and nitrate are the major species in the spectrum while other species (if any) have a minor effect in the spectral configuration in that range. So, this method is well suited for typical drinking water. 

The polynomial regression model performance can be further improved by adding other water quality parameters (if available) as predicting variables. Overall, the method is the same except the number of predictor variables is increased to obtain a better fit.

**Figure A4:**
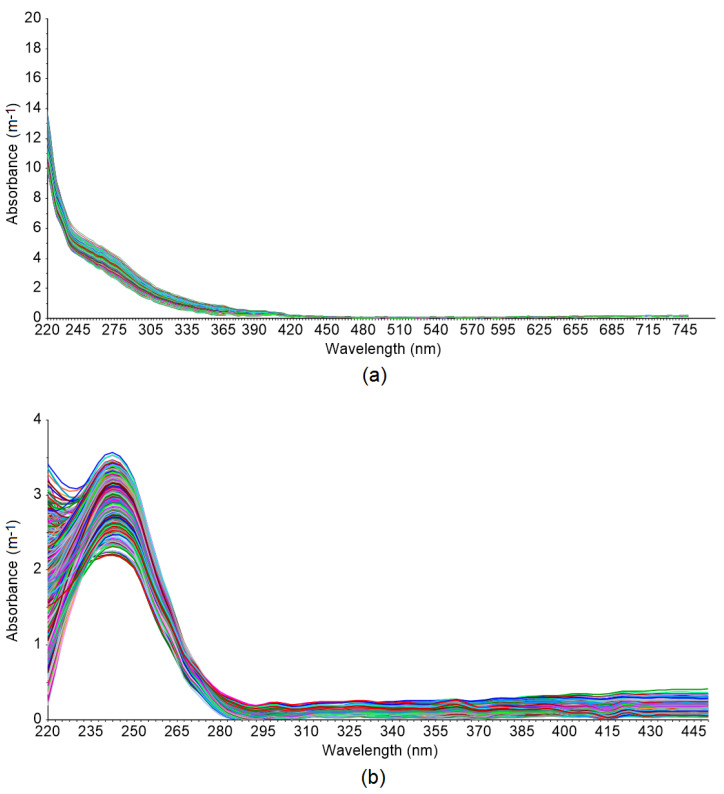
(**a**) Estimated pre chloraminated spectra from DOC and NO_3_-N using polynomial regression and (**b**) estimated NH2Cl spectra (these spectra were obtained by subtracting Figure A4a spectra from Figure A2b spectra. For better visibility, up to 445 nm is shown here. The remaining region is flat).

The DOC and NO_3_-N compensated spectra are presented in Figure 9e, which are identical to the typical monochloramine spectra presented in Figure 8a. Wavelengths of only 220 to 330 nm are shown in the figure as the remaining region of the spectra is comparatively flat (full-wavelength spectra are given in Appendix A, Figure A4). The peak absorbance appeared at about the 245-nm wavelength, which is characteristic of a typical monochloramine spectrum. Some portion of the spectra starting from the 280-nm wavelength shows negative absorbance, which is subjected to estimation error by the polynomial model and corresponding arithmetic subtraction. A baseline correction was applied using a linear offset method while developing the SVR model with these spectral data.

### 3.3. SVR Model Fitting

**Figure 10:**
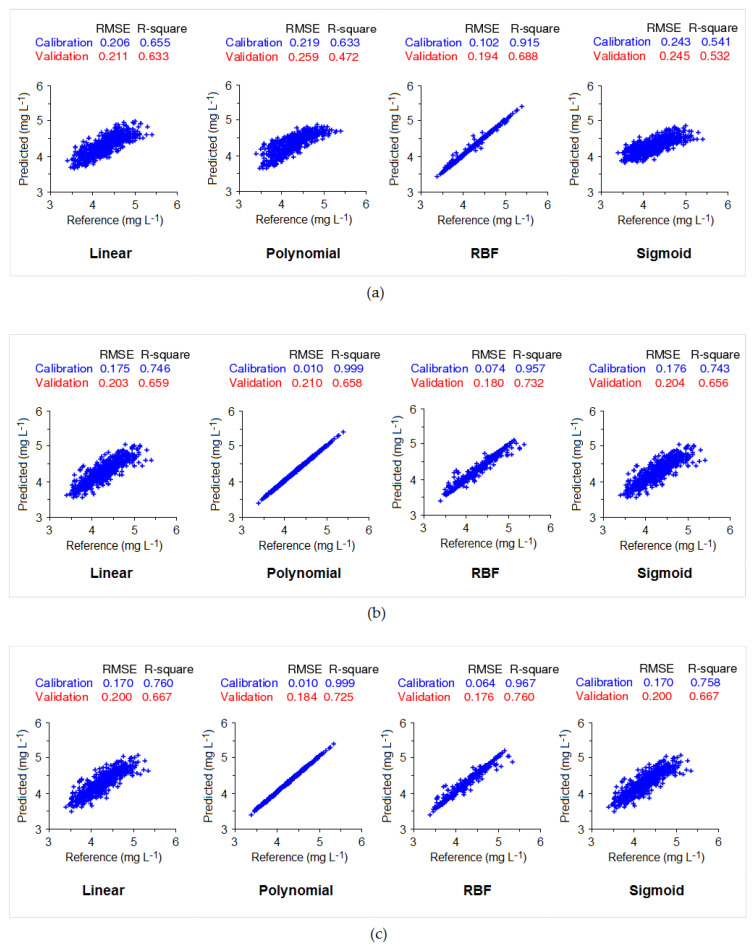
Support vector regression (SVR) performance in model training for different kernel functions: (**a**) Uncompensated spectra (**b**) Particle compensated spectra; and (**c**) particle, organic, and nitrate compensated spectra.

Using both particle compensated and uncompensated spectra, the SVR model was developed. The ε value was set to 0.01, which means data points that fall within this margin will be considered insensitive. The model training accuracy of the uncompensated spectra is presented in Figure 10a while the particle compensated spectra are presented in Figure 10b. The term “reference” in the *x*-axis in the figure means the observed monochloramine concentration data from the amperometric analyser. It can be seen in Figure 10a that for uncompensated spectra, the best agreement between both data sets was achieved by using the RBF kernel with an R-square value of 0.915 and RMSE of 0.102. In contrast, the other kernels do not indicate a reasonable level of agreement between the reference and predicted values. Figure 10b shows a good level of model training performance in the particle compensated spectra using the polynomial and RBF kernel, with R-square values of 0.999 and 0.957, respectively. The RMSE values with the polynomial and the RBF kernel are 0.010 and 0.074, respectively, indicating a deficient error in the model training. For the linear and sigmoid kernels, data points in the graph are more sparsely fitted, with a comparatively high RMSE and lower R-square values than polynomial and RBF kernels.

Performance during the cross-validation was comparatively weaker than that during the model training phase in both cases. For uncompensated spectra, the RBF kernel showed a relatively better performance and encountered lower error than other kernels, with an R-square value of 0.688 and RMSE of 0.194. In contrast, particle compensated spectra showed a better performance for all kernels, with the highest accuracy obtained by the RBF kernel, achieving an R-square value of 0.732, and RMSE value of 0.180.

The SVR model training performance using particle, organic, and nitrate compensated spectra combined is presented in Figure 10c. Here, the polynomial kernel shows a near perfect fit in model training with an R-square of 0.999 and RMSE of 0.010, while the RBF kernel shows a comparatively lower performance with an R-square of 0.967 and RMSE of 0.064. The linear and sigmoid kernels did not indicate a similarly good performance in the model training phase. In the cross-validation phase, RBF has the highest performance with an R-square of 0.760 and RMSE of 0.176 while the polynomial kernel has the second most performance with an R-square of 0.725 and RMSE of 0.184. The analysis of the standard deviation indicates that the level of precision by the model was ±0.1 mg L^‒1^.

## 4. Discussion

### 4.1. Comparison of Model Performance

**Figure 11:**
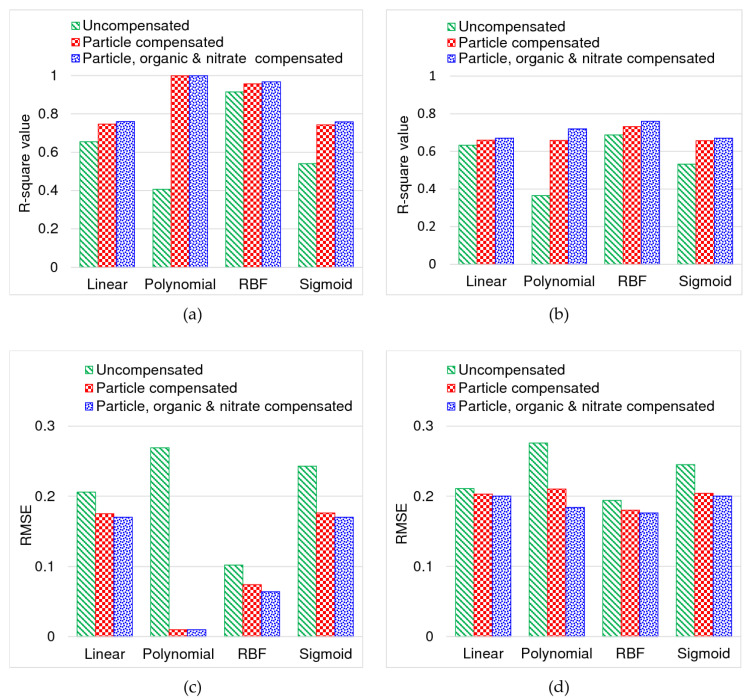
Comparison of SVR modelling performance: (**a**) R-square in model training, (**b**) R-square in cross-validation, (**c**) RMSE in model training, and (**d**) RMSE in cross-validation.

Figure 11 compares the SVR modelling performance visually with the help of a column chart for the above three cases: (i) uncompensated or original chloraminated water spectra; (ii) particle compensated spectra; and (ii) particle, organic, and nitrate compensated spectra. Numeric data for these comparisons are provided in Appendix B, Table A2, and Table A3. The R-square and RMSE values in model training and cross-validation indicated that particle, organic, and nitrate compensated spectra with the RBF kernel function can better represent the monochloramine residual concentration. Although the polynomial kernel showed a better fitting with the training data, its performance in cross-validation was relatively lower with error relatively higher than the RBF kernel. Considering the cross-validation performance, RBF appeared to be the most appropriate kernel function. From the figure, it is also evident that uncompensated or original spectra cannot be satisfactorily used in determining the monochloramine residual concentration.

The above procedure can be implemented more efficiently by reducing the sample size. Most SVR algorithms require the provision of training samples in a single batch [38]. A new model will require that every time a new sample is added or removed from the training set. Here, three months of data were used in a single batch, which was huge for the purpose of relating spectral features with monochloramine data. Once the appropriate kernel function is determined, recent observations can be used instead of the whole data to train the model. This will significantly reduce the SVR model runtime.

### 4.2. Limitations of the Research

During the chloramination process, along with monochloramine, some dichloramine and trichloramine can form. Control of the chloramination process means that the formation of dichloramine and trichloramine is minimal and is assumed to have negligible interference on the monochloramine spectrum. This research only focused on spectral detection of monochloramine while dichloramine and trichloramine impacts were out of the scope of this paper.

The spectrophotometer’s built-in tool was used to complete the particle compensation. However, different manufacturers use different particle compensation algorithms in their instrument. This should be explored as the modelling accuracy greatly depends on particle compensation.

The WTP-post chloramination pH was relatively stable, with an average of 8.67 and standard deviation of 0.27, so no spectral shifting was considered. However, in cases of online monitoring where the pH of incoming water significantly varies with time, a pH compensation can be considered to correct spectral shifting. 

The quality of drinking water varies from place to place. During the study period, the concentration of DOC ranged between 1.7 and 2.7 mg L^‒1^ while the NO_3_-N concentration ranged between 0.1 and 0.4 mg L^‒1^ and the monochloramine concentration ranged between 3.0 and 5.5 mg L^‒1^. Hence, the spectral compensation and the associated SVR model works well within this range. Beyond this range, the modelling accuracy may differ.

## 5. Conclusions

Effective spectral online detection of drinking water disinfectant (monochloramine) was proposed in this paper. The Tailem Bend drinking water treatment plant in South Australia, which currently uses an amperometric online chlorine analyser to monitor monochloramine residual, was selected as the case study. An online UV-Vis spectrophotometer probe was installed at the WTP to gather spectral water quality information. Absorbance data at various wavelengths were treated in several stages to ensure quality and PCA was used to extract features from these data. In developing the machine learning model, these spectral features were considered as predictor or independent variables while the amperometric analyser data were used as the response or dependent variable.

The SVR algorithm with four different kernel functions: (i) linear, (ii) polynomial, (iii) RBF, and (iv) sigmoid, was considered to determine the best-fitting model. The R-square and RMSE in model training and cross-validation indicated that RBF has better accuracy over other kernels in determining the monochloramine concentration using both compensated and uncompensated spectra. In specific, particle compensated spectra showed better model fitting and lower error than uncompensated spectra. Additionally, compensation for organic (DOC) and nitrate (NO_3_-N) was shown to improve the modelling performance. Finally, the following conclusions can be drawn:

- Machine learning with UV-Vis spectrometry can be used in online detection of monochloramine residual;
- The choice of the kernel function has a high impact in modelling performance, particularly, RBF kernel has better accuracy for non-linear mapping of spectral data; and
- Particle compensation and the newly introduced organic and nitrate compensation improves modelling accuracy.

## Appendix B

**Table A1:** R-square and RMSE values in polynomial model fitting using NO_3_-N and DOC and absorbance at various wavelengths.

Wavelength (nm)	R-Square	RMSE	Wavelength (nm)	R-Square	RMSE	Wavelength (nm)	R-Square	RMSE
220	0.9164	0.3275	397.5	0.7522	0.0186	575	0.0707	0.0048
222.5	0.9284	0.2633	400	0.7359	0.0173	577.5	0.2760	0.0054
225	0.9487	0.1831	402.5	0.7541	0.0156	580	0.1558	0.0041
227.5	0.9661	0.1244	405	0.7327	0.0150	582.5	0.2685	0.0051
230	0.9735	0.0976	407.5	0.6383	0.0167	585	0.2684	0.0056
232.5	0.9819	0.0720	410	0.7127	0.0142	587.5	0.1004	0.0052
235	0.9887	0.0516	412.5	0.7237	0.0144	590	0.3123	0.0052
237.5	0.9900	0.0452	415	0.6850	0.0158	592.5	0.3177	0.0058
240	0.9892	0.0446	417.5	0.7042	0.0182	595	0.4229	0.0052
242.5	0.9881	0.0449	420	0.7294	0.0148	597.5	0.2581	0.0054
245	0.9882	0.0440	422.5	0.6889	0.0109	600	0.1275	0.0049
247.5	0.9889	0.0423	425	0.6698	0.0104	602.5	0.2366	0.0054
250	0.9904	0.0396	427.5	0.4827	0.0106	605	0.2209	0.0045
252.5	0.9932	0.0339	430	0.6553	0.0077	607.5	0.2487	0.0052
255	0.9947	0.0303	432.5	0.6268	0.0089	610	0.2179	0.0059
257.5	0.9954	0.0284	435	0.6094	0.0080	612.5	0.3086	0.0059
260	0.9960	0.0268	437.5	0.6328	0.0066	615	0.3980	0.0073
262.5	0.9966	0.0250	440	0.5436	0.0064	617.5	0.2363	0.0056
265	0.9976	0.0212	442.5	0.5335	0.0054	620	0.3605	0.0063
267.5	0.9985	0.0168	445	0.4764	0.0044	622.5	0.2977	0.0056
270	0.9989	0.0141	447.5	0.3530	0.0050	625	0.3081	0.0063
272.5	0.9995	0.0097	450	0.2955	0.0053	627.5	0.3099	0.0075
275	0.9998	0.0052	452.5	0.4921	0.0039	630	0.2489	0.0074
277.5	1.0000	0.0004	455	0.4920	0.0041	632.5	0.2515	0.0071
280	0.9997	0.0065	457.5	0.2891	0.0039	635	0.1987	0.0081
282.5	0.9988	0.0123	460	0.3920	0.0037	637.5	0.2724	0.0068
285	0.9983	0.0139	462.5	0.4002	0.0043	640	0.2909	0.0066
287.5	0.9984	0.0126	465	0.3283	0.0047	642.5	0.3857	0.0085
290	0.9982	0.0124	467.5	0.4024	0.0050	645	0.4697	0.0079
292.5	0.9966	0.0157	470	0.2940	0.0049	647.5	0.4383	0.0077
295	0.9930	0.0210	472.5	0.2715	0.0054	650	0.3744	0.0094
297.5	0.9896	0.0240	475	0.1537	0.0058	652.5	0.2757	0.0100
300	0.9883	0.0238	477.5	0.1678	0.0062	655	0.4766	0.0079
302.5	0.9883	0.0225	480	0.1382	0.0052	657.5	0.4586	0.0085
305	0.9883	0.0211	482.5	0.4923	0.0052	660	0.3474	0.0079
307.5	0.9873	0.0206	485	0.7846	0.0065	662.5	0.3433	0.0088
310	0.9862	0.0202	487.5	0.7375	0.0065	665	0.4337	0.0089
312.5	0.9858	0.0192	490	0.5191	0.0057	667.5	0.2991	0.0097
315	0.9836	0.0197	492.5	0.6808	0.0050	670	0.2686	0.0117
317.5	0.9788	0.0216	495	0.5347	0.0048	672.5	0.3862	0.0086
320	0.9735	0.0231	497.5	0.6630	0.0047	675	0.5706	0.0111
322.5	0.9686	0.0240	500	0.6226	0.0058	677.5	0.5329	0.0099
325	0.9663	0.0237	502.5	0.4703	0.0057	680	0.4096	0.0102
327.5	0.9629	0.0238	505	0.1710	0.0048	682.5	0.4444	0.0106
330	0.9591	0.0240	507.5	0.4694	0.0044	685	0.4717	0.0113
332.5	0.9573	0.0233	510	0.3895	0.0050	687.5	0.4326	0.0114
335	0.9540	0.0232	512.5	0.4054	0.0049	690	0.4302	0.0113
337.5	0.9508	0.0229	515	0.3332	0.0060	692.5	0.3367	0.0107
340	0.9484	0.0223	517.5	0.2726	0.0051	695	0.4827	0.0100
342.5	0.9475	0.0216	520	0.2026	0.0045	697.5	0.5418	0.0121
345	0.9425	0.0217	522.5	0.3525	0.0040	700	0.5341	0.0108
347.5	0.9301	0.0232	525	0.5074	0.0047	702.5	0.4690	0.0099
350	0.9232	0.0234	527.5	0.4303	0.0049	705	0.4626	0.0120
352.5	0.9174	0.0236	530	0.6237	0.0053	707.5	0.4440	0.0133
355	0.9025	0.0253	532.5	0.5686	0.0061	710	0.3627	0.0114
357.5	0.8788	0.0281	535	0.5214	0.0073	712.5	0.3789	0.0126
360	0.8600	0.0301	537.5	0.6233	0.0051	715	0.3239	0.0143
362.5	0.8398	0.0315	540	0.3470	0.0053	717.5	0.3650	0.0122
365	0.8335	0.0304	542.5	0.3967	0.0046	720	0.4993	0.0127
367.5	0.8570	0.0266	545	0.3976	0.0058	722.5	0.5222	0.0124
370	0.8721	0.0234	547.5	0.4221	0.0063	725	0.4412	0.0124
372.5	0.8718	0.0214	550	0.5608	0.0056	727.5	0.4156	0.0129
375	0.8614	0.0209	552.5	0.3943	0.0058	730	0.4511	0.0135
377.5	0.8361	0.0221	555	0.1816	0.0048	732.5	0.4651	0.0140
380	0.8178	0.0226	557.5	0.1285	0.0042	735	0.4389	0.0143
382.5	0.8214	0.0216	560	0.1987	0.0045	737.5	0.3717	0.0140
385	0.8174	0.0204	562.5	0.1783	0.0044	740	0.3918	0.0147
387.5	0.7958	0.0200	565	0.3906	0.0042	742.5	0.4228	0.0175
390	0.7743	0.0203	567.5	0.4372	0.0055	745	0.4344	0.0169
392.5	0.7586	0.0208	570	0.2402	0.0047	747.5	0.3577	0.0193
395	0.7567	0.0202	572.5	0.0906	0.0045	-		

**Table A2:** SVR modelling performance in terms of RMSE and R-square for different kernel functions in model training.

**Uncompensated or Raw Spectra**		
Kernel Function Type	RMSE	R-square
Linear kernel	0.206	0.655
Polynomial kernel	0.219	0.633
RBF kernel	0.028	0.994
Sigmoid kernel	0.243	0.541
**Particle Compensated Spectra**		
Kernel Function Type	RMSE	R-square
Linear kernel	0.175	0.746
Polynomial kernel	0.010	0.999
RBF kernel	0.074	0.957
Sigmoid kernel	0.176	0.743
**Particle, DOC and NO_3_-N Compensated Spectra**		
Kernel Function Type	RMSE	R-square
Linear kernel	0.170	0.760
Polynomial kernel	0.010	0.999
RBF kernel	0.064	0.967
Sigmoid kernel	0.170	0.758

**Table A3:** SVR modelling performance in terms of RMSE and R-square for different kernel functions in cross-validation.

**Uncompensated or Raw Spectra**		
Kernel Function Type	RMSE	R-square
Linear kernel	0.211	0.633
Polynomial kernel	0.259	0.472
RBF kernel	0.199	0.680
Sigmoid kernel	0.245	0.532
**Particle Compensated Spectra**		
Kernel Function Type	RMSE	R-square
Linear kernel	0.203	0.659
Polynomial kernel	0.210	0.658
RBF kernel	0.180	0.732
Sigmoid kernel	0.204	0.656
**Particle, DOC and NO_3_-N Compensated Spectra**		
Kernel Function Type	RMSE	R-square
Linear kernel	0.200	0.670
Polynomial kernel	0.184	0.720
RBF kernel	0.176	0.760
Sigmoid kernel	0.200	0.670
